# Macular dynamics and visual acuity prognosis in retinal vein occlusions - ways to connect

**DOI:** 10.22336/rjo.2023.51

**Published:** 2023

**Authors:** Diana-Maria Dărăbuș, Cristina-Patricia Pac, Cosmin Roşca, Mihnea Munteanu

**Affiliations:** * Department of Ophthalmology, “Victor Babeş” University of Medicine and Pharmacy, Timişoara, Romania; ** Oculens Clinic, Cluj Napoca, Romania

**Keywords:** retinal vein occlusions, visual acuity prognosis, central macular thickness, hyperreflective foci, foveal intraretinal hemorrhage, intravitreal therapy, ellipsoid zone disruption

## Abstract

**Background and Objectives:** This study aimed to establish possible connections between macular dynamics, various macular features, and visual acuity prognosis among patients with retinal vein occlusions.

**Materials and Methods:** This study included 85 patients with central retinal vein occlusions (CRVO) and 26 with branch retinal vein occlusions (BRVO). We assessed macular features such as central macular thickness (CMT), foveal intraretinal hemorrhage (IRH), the presence and distribution of hyperreflective foci (HF), ellipsoid zone (EZ) disruption, inner retinal layer disorganization (DRIL), and posterior vitreous detachment (PVD), as well as their dynamics over one year of observation and their impact on final visual acuity prognosis, depending on the type of occlusion.

**Results:** Best corrected visual acuity (BCVA) evolution is statistically significant regarding groups of age and type of occlusion and insignificant regarding gender. The best response to intravitreal treatment, quantified as a decrease in CMT, was registered after the first intravitreal injection. Connecting a decrease in CMT with BCVA improvement, we did not register a statistically significant correlation in the CRVO group, only in BRVO cases. The study results showed that complete PVD plays a significant positive role in decreasing CMT and BCVA improvement in cases of CRVO. Our study revealed that no matter the type of occlusion, the presence of foveal IRH will have a negative impact on the BCVA outcome. Statistically significant differences have been noted only for the evolution of visual acuity in non-ischemic CRVO cases, in correlation with the presence of EZ disruption. Outer retinal layer HF has proved to be a predictive factor for poor visual acuity outcomes.

**Conclusions:** The most important non-imaging predicting factors regarding BCVA after retinal vein occlusions are age and baseline BCVA. CMT’s dynamics still establish a weak connection with visual acuity fluctuations. The presence of foveal IRH, outer retinal layer HF, and foveal EZ disruption has a negative impact on visual acuity outcomes.

**Abbreviations: **CRVO = central retinal vein occlusions, BRVO = branch retinal vein occlusions, CMT = central macular thickness, IRH = foveal intraretinal hemorrhage, HF = hyperreflective foci, EZ = ellipsoid zone disruption, DRIL = inner retinal layer disorganization, PVD = posterior vitreous detachment, BCVA = best corrected visual acuity, OCT = optical coherence tomography, BCVA Ti = best corrected visual acuity at first, BCVA Tf = best corrected visual acuity after one year, NR of IVI = number of intravitreal injections, SD = standard deviation, M = male, F = female, CMT Ti = central macular thickness at first, CMT T1 = central macular thickness after first injection, CMT T3 = central macular thickness after 3 injections, CMT Tf = central macular thickness after one year

## Introduction

Retinal vein occlusions represent one of the most common retinal vascular pathologies [**1**] and a common cause of loss of vision in older patients [**2**]. Studying this condition from a broad perspective is necessary, taking into consideration associated risks in general [**3**]. Several studies have already shown that the type of occlusion is crucial for visual acuity prognosis [**4**], and also the age of development, with older patients being associated with poorer gain in visual acuity after treatment [**5**]. These are non-imaging predictive factors for visual acuity prognosis. On the other hand, although it has been considered a reference point, no significant and reliable results have been obtained to establish a connection between central macular thickness (CMT) and visual acuity prognosis [**6**]. Several macular features have received a lot of interest in recent decades as possible prognosis factors: hyperreflective foci, foveal intraretinal hemorrhage, ellipsoid zone disruption, retinal inner layers integrity, and vitreomacular adhesion [**7**]. Some are already considered biomarkers of poor prognosis, others are viewed as biomarkers of weak treatment response, and some are still under question regarding their relevance in retinal vein occlusions.

In this retrospective study, we aimed to underline possible connections between macular dynamics regarding CMT and visual acuity prognosis. Secondly, we aimed to observe how the already presented macular features could affect one another in time and their specific ways of influencing the visual acuity outcome.

## Materials and Methods

*Study Design and Population*

This study was retrospective and was conducted at Professor Munteanu Mihnea Eye Clinic in Timişoara, Romania, from September 2020 to September 2022. 111 eyes of 111 patients diagnosed with unilateral retinal vein occlusion were included in the study, among whom 85 had central retinal vein occlusion, 26 had branch retinal vein occlusion and none had hemiretinal vein occlusion. The follow-up period was 12 months. All patients signed informed consent forms according to the institutional guidelines. This study was part of a doctoral dissertation that was approved by the Ethics Committee from “Victor Babes University of Medicine and Pharmacy Timişoara”, record number 68/2020.

Age and gender were registered at first, with visual acuity being analyzed in correlation with these two factors (three groups were formed: under 40 years old, between 40 and 60 years old, and over 60 years old). Independently, patients included in the study were split into three groups, depending on the type of occlusion (branch retinal vein occlusions, non-ischemic central retinal vein occlusion, or ischemic central retinal vein occlusion), and analyzed depending on age and gender. Intravitreal treatment was performed using bevacizumab alone or bevacizumab with triamcinolone, depending on the case, retinal aspect, and after the following considerations: monthly injections in the first 3 months followed by a treat-and-extend-type treatment, depending on the retinal response. Any switch in the therapeutic agent during the first year of observation was not considered at that time because, in comparison with bevacizumab, much higher out-of-pocket costs were correlated with other available anti-VEGF agents (aflibercept, brolucizumab), because no possibility for reimbursement was available in the Romanian healthcare system.

Firstly, central macular thickness (CMT) was assessed before intravitreal treatment (CMT Ti), after the first intravitreal injection (CMT T1), after 3 intravitreal injections (CMT T3), and after one year of supervision (CMT Tf). The dynamics of CMT were compared depending on the type of occlusion and correlated with the visual acuity evolution. Secondly, the retinal response observed a decrease in CMT after intravitreal injections and visual acuity improvement was assessed according to the presence or absence of posterior vitreous detachment (PVD). Thirdly, the presence and, in some cases, the location of the following macular features were assessed during the time of observation and analyzed as predictive factors regarding visual acuity evolution: the presence of hyperreflective foci (outer retinal layers HF or inner retinal layers HF), foveal intraretinal hemorrhage (foveal IRH), retinal inner layer disorganization (DRIL), and ellipsoid zone (EZ) disruption (analyzed separately, foveal EZ disruption and parafoveal EZ disruption zones).

*Data Collection*

The patients included in this study were those with BRVO or CRVO who were registered in the study center and completed all the required follow-ups, investigations, and treatments during at least one year of observation. The exclusion criteria were patients who were non-compliant with the treatment, follow-ups or investigations, patients with an allergy to any substance that was used, pregnancy, mixed occlusions, associated diabetes (due to possible diabetic neuropathy or neovascular implication), associated unoperated cataracts, associated age-related macular degeneration, vitreous hemorrhage, bad quality of images because of strong eye movements, and any other previous intravitreal treatment [**8**].

The principles of selecting the exclusion criteria were targeting factors that impacted visual acuity or the visualization of the retinal aspect (cataract, vitreous hemorrhage at first or at the final assessment) and factors that independently produced alterations in the macula (diabetes, age-related macular degeneration), mainly because the purpose of this study was to find correlations between these two aspects. Therefore, 3 cases of vitreous hemorrhage at the moment of presentation and 1 case of vitreous hemorrhage at the final assessment were excluded from the study. Moreover, 67 of the patients included in the study were pseudophakic from the moment of presentation, 24 with no significant lens opacities at the moment of presentation required cataract surgery during the monitoring period (due to the development of significant opacities after intravitreal treatment), with surgery being performed before the final assessment, and 20 patients did not develop significant lens opacities and did not require cataract surgery.

During the monitoring time, among all 31 patients with an ischemic type of occlusion, 4 patients developed neovascular glaucoma in the first 6 months and 5 patients between 6 and 12 months. In all cases, intraocular pressure control was still possible with antiglaucomatous treatment, with no need for additional invasive therapy. After intravitreal triamcinolone, 14 cases of high intraocular pressure were registered and all responded well to topical medication and did not represent a clinical issue. After 12 months of treatment and observation, 3 patients presented resistant cystoid macular edema, needing a new anti-VEGF treatment.

The following data were collected: visual acuity at the first presentation, at every checkup, and after one year of observation (measured by a best-corrected-visual-acuity-certified technician), approximate time of occlusion, age, gender, and number and type of intravitreal injections during the follow-up period (intravitreal injections with bevacizumab or bevacizumab with triamcinolone). A slit lamp examination for the anterior pole and a dilated pupil fundus examination was performed during the first examination, at every check-up, and of course during the final assessment.

The type of occlusion was identified in a few cases using the HD ultra-widefield fundus imaging module from Clarus 700 Zeiss, showing extensive intraretinal hemorrhages associated with the tortuosity of retinal vessels, associated with BCVA worse than 1.0 logMAR. Meanwhile, in most cases, by performing fluorescein angiography (using Fluorescite 10% dozes of 5 mL from Alcon Laboratories INC, Fort Worth, Texas 76134 USA.) based on the fluorescein angiography module from Clarus 700, areas of retinal capillary nonperfusion greater than 10-disc areas in diameter were correlated with an ischemic type of occlusion [**9**]. The presence of foveal IRH was assessed using fundus photographs from Clarus 700 Zeiss - HD Ultra-widefield Fundus Imaging, the assessment being performed by a human grader and confirmed by optic coherence tomography. The fovea was considered to be the area located 2.5-disc radii temporal and slightly inferior to the optic disc, being 1-disc diameter in size. 

Several macular features were assessed using Cirrus HD-OCT-High Definition Optical Coherence Tomography (model 5000, software version 100, Carl Zeiss Meditec, Inc. Dublin, CA, USA), by a previously certified, independent and blinded grader. Ellipsoid zone (EZ) integrity was assessed by additionally using En-Face analysis of the macular cube 512 × 128, with disruption zones being analyzed and, depending on their location, divided into foveal (1 mm diameter foveal zone) or parafoveal disruptions. Patients with both foveal and extrafoveal disruptions were included in foveal group disruptions.

Hyperreflective foci were small dot-shaped lesions that proved to have a reflectivity at least as high as the retinal pigment epithelium, with a well-defined outline. To distinguish between HF and other hyper-reflective conditions (for example, retinal exudates, microaneurysms, or hemorrhages), HF dots were under 30 µm in size, with no posterior shadowing, located in the inner or outer retinal layers and with similar reflectivity to the retinal nerve fiber layer [**10**]. After the identification of HF, the predominant layer was assessed and all cases were split regarding the predominant location of HF: the inner retinal layer, outer retinal layer, and, of course, no identified HF. 

Macular thickness was analyzed by using Macular Cube 512 × 128, the default scan for such cases, which created a 6 mm-diameter square grid with 128 horizontal scan lines, each formed by 512 A-scans and a central horizontal HD B-scan. Raster scans (5 Line Raster or HD 21 line) were used for DRIL identification and the identification of the presence and distribution of HF. Posterior vitreous detachment was analyzed firstly with ultrasonography and biomicroscopy and sometimes using macular OCT.

*Statistical Analysis*

 Statistical calculations were performed using SPSS (Version 20, IBM), and Microsoft Excel (2016, Microsoft Corporation, Redmond, WA, USA). For the variables of concern, elements of descriptive statistics were computed (mean, standard deviation), and statistical tests were applied to determine the significant differences (ANOVA, Kruskal-Wallis, and Mann-Whitney). The graphical representations that were used were bar diagrams and error bar diagrams (mean and 95% confidence interval for mean).

## Results

*Characteristics of the Participants*

The study included 111 patients with unilateral retinal vein occlusion, meaning 85 with central retinal vein occlusion (CRVO) and 26 with branch retinal vein occlusion (BRVO). Among them, 57 were men and 54 were women. The mean age for those who developed ischemic CRVO was 66.25 ± 10.576, for those who developed non-ischemic CRVO it was 69.148 ± 14.788, and for those with BRVO, it was 57 ± 9.365 (**Table 1**).

**Table 1:** Baseline characteristics of the patients

Baseline Characteristic			CRVO		BRVO
			Ischemic	Non-Ischemic	
No. of eyes/patients		No	31	54	26
		%	27.93	48.65	23.42
Age			66.258 ± 10.576	69.148 ± 14.788	57 ± 9.365
Men		No	19	27	11
		%	17.12	24.32	9.37
Women		No	12	27	15
		%	10.81	24.32	13.51
BCVA Ti logMAR	>1.00	No	31	24	3
		%	27.93	21.62	2.70
	1.00-0.5	No	0	26	3
		%	0	23.42	2.70
	<0.5	No	0	4	20
		%	0	3.60	12.02
BCVA Tf logMAR	>1.00	No	31	6	0
		%	27.93	5.41	0
	1.00-0.5	No	0	24	3
		%	0	21.62	20.72
	<0.5	No	0	24	23
		%	0	21.62	23.42
NR. of IVI	5	No	16	7	26
		%	0.14	0.06	0.23
	7	No	9	26	0
		%	8.11	23.42	0
	8	No	6	21	0
		%	5.41	18.92	0
CRVO = central retinal vein occlusion, BRVO = branch retinal vein occlusion, BCVA Ti = best corrected visual acuity at first, BCVA Tf = best corrected visual acuity after one year, NR of IVI = number of intravitreal injections.					

 All patients with ischemic CRVO presented with a logMAR visual acuity worse than 1.0, which stayed that way even after one year. Twenty-four patients with non-ischemic CRVO presented with logMAR visual acuity worse than 1.0, 26 had a visual acuity between 1.0 and 0.5 logMAR and only 6 had a visual acuity greater than 0.5 logMAR. Nevertheless, in this group, only 6 patients still had a visual acuity lower than 1.0 logMAR after one year, while 24 had a visual acuity between 1.0 and 0.5 logMAR, and 24 had a visual acuity of more than 0.5 logMAR. Regarding the BRVO group, after one year, most of the patients had a visual acuity better than 0.5 logMAR. These changes underline the importance of BCVA at first and the type of occlusion in visual acuity prognosis. Moreover, all patients with BRVO completed an intravitreal treatment of five injections during the first year of monitoring, while those with non-ischemic CRVO needed the highest number of intravitreal injections. Patients with ischemic CRVO registered a lower need for this kind of treatment, especially due to their tendency for macular atrophy (**Table 1**).

*Age and Gender in Connection with Visual Acuity Prognosis*

*Gender*

This study included 57 male patients and 54 female patients, with a slightly higher mean logMAR BCVA registered among female patients (1.1481 ± 0.6892 at first and 0.7694 ± 0.6515 after one year) in comparison with male patients (1.2474 ± 0.5471 at first and 0.9895 ± 0.6851 after one year). Differences between BCVA regarding gender were statistically insignificant at first (ANOVA test, F = 0.710, p = 0.401 > 0.05) and after one year (ANOVA test, F = 2.99, p = 0.086 > 0,05). Moreover, analyzing BCVA from an evolutive perspective, no statistically significant differences were noted concerning gender (ANOVA test, F = 1.742, p = 0.190 > 0.05).

*Age*

The best corrected visual acuity was analyzed firstly from a descriptive point of view between the three groups of age, at first and after one year. Afterward, BCVA was assessed from an evolutive point of view, firstly depending on the group of age and secondly depending on the group of age and gender. The best logMAR BCVA prognosis was registered in patients between 40 and 60 years old, no matter the type of occlusion, where at first, the mean logMAR BCVA was 0.7300 ± 0.5790, better than the logMAR BCVA at first in the under 40 years old group (0.7891 ± 0.4739), and after one year of treatment (0.4583 ± 0.5847 for the 40-60 years old group in comparison with 0.4589 ± 0.7593 for under 40 years old group). Modest results were registered in patients over 60 years old, who presented the lowest logMAR mean BCVA at first (1.4093 ± 0.5350) and after one year (1.0387 ± 0.6484).

From a descriptive point of view, logMAR BCVA registered no statistically significant differences at first, between the under 40 years old group and the 40-60 years old group (Mann-Whitney test, U = 63, p = 0.248> 0.05), and even after one year (Mann-Whitney test, U = 79.5, p = 0.651 > 0.05). Nevertheless, statistically significant differences were registered in comparing the under 40 years group of patients with the over 60 years old group at first (Mann-Whitney test, U = 126, p = 0.03 < 0.05) and after one year of treatment (Mann-Whitney test, U = 127, p = 0.047 < 0.05) and also the between 40 and 60 years old group of patients and more than 60 years old group at first (Mann-Whitney test, U = 43, p = 0.000 < 0.05) and also after one year (Mann-Whitney test, U = 553, p = 0.000 < 0.05).

From an evolutive perspective and regarding age, the evolution of BCVA presented statistically significant differences (Kruskal-Wallis Test, X2 = 14.244, p = 0.001 < 0.05). In one year, the under 40 years old group, in comparison with the over 60 years old group, registered statistically significant differences (Mann-Whitney Test, U = 196.00, p = 0.023 < 0.05), and no statistically significant differences in comparison with the 40-60 years old group (Mann-Whitney Test, U = 45.50, p = 0.057 > 0.05). Nevertheless, also from an evolutive perspective, statistically significant differences were registered when comparing the over 60-year-old group of patients with the 40-60-year-old group (Mann-Whitney Test, U = 605.50, p = 0.000 < 0.05).

**Fig. 1:**
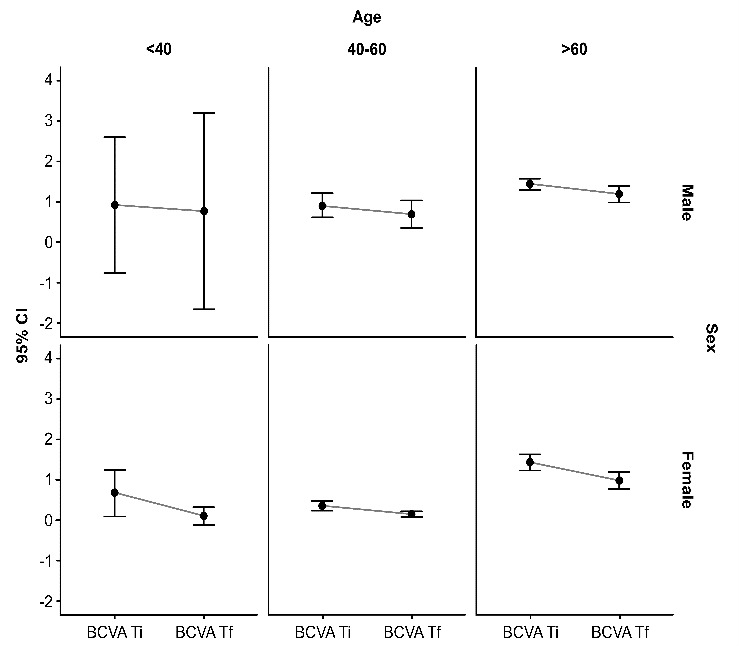
Visual acuity in correlation with age and gender

Judging from the evolutive perspective and also depending on gender, the greatest progression was registered in female patients, for all three age groups (an increase of 0.2524 ± 0.128 for the under 40 years old group, an increase of 0.4635 ± 0.1533 for the 40-60 years old group and an increase of 1.2186 ± 0.5589 for the over 60 years old group), and the smallest increase in BCVA was registered in patients over 60 years old, no matter the gender (1.2717 ± 0.5726 male cases and 1.2186 ± 0.5589 female cases) (**Table 2**, **Fig. 1**).

**Table 2:** Statistical differences between the evolution of logMAR BCVA depending on groups of age and gender

Evolution of BCVA	<40	40-60	>60	U(*p*)	U(*p*)	U(*p*)
	(Mean ± SD)	(Mean ± SD)	(Mean ± SD)	<40 vs. 40-60	40-60 vs. >60	<40 vs. >60
M	1.0153 ± 0.08528	0.9708 ± 0.60385	1.2717 ± 0.57264	Kruskal-Wallis Test p = 0.077		
F	0.2524 ± 0.128	0.4635 ± 0.15332	1.2186 ± 0.55892	6 (0.008)	67.5 (0.000)	6 (0.000)
BCVA = best corrected visual acuity, SD = standard deviation, M = male, F = female.						

*Central Macular Thickness Variations in Connection with Visual Acuity Outcomes*

*CMT under Intravitreal Therapy and BCVA Outcomes*

 Taking into consideration the presented types of retinal venous occlusion, the evolution of the central macular thickness was assessed, from the moment of presentation to after the first injection, after three intravitreal injections, and after one year (pointing out the fact that after the first three injections, all the patients continued intravitreal therapy as prescribed by the doctor, depending on the macular dynamics and as presented at the very beginning).

 • CRVO (ischemic and non-ischemic)

 No statistically significant differences were found between ischemic and non-ischemic central retinal vein occlusions, concerning central macular thickness at first (ANOVA test, F = 0.56, p = 0.455 > 0.05), but statistically significant differences were registered regarding the response to intravitreal therapy after the first injection (ANOVA test, F = 14.57, p = 0.000 < 0.05), after three doses (ANOVA test, F = 14.39, p = 0.000 < 0.05) and after one year (ANOVA test, F = 26.22, p = 0.000 < 0.05) (**Table 3**).

**Table 3:** Statistical differences between the evolution of logMAR BCVA depending on groups of age and gender

	CRVO		
	NO (Mean ± SD)	YES (Mean ± SD)	F(p) No vs. YES
CMT Ti	566.0556 ± 199.8242	596.6129 ± 140.8036	0.56(0.455)
CMT T1	424.1481 ± 199.8242	305.9032 ± 85.69067	14.57 (0.000 *)
CMT T3	324.685 ± 121.1249	238.87 ± 44.33414	14.39 (0.000 *)
CMT Tf	292.537 ± 94.43	204.161 ± 21.66579	26.22 (0.000 *)
CMT Ti = central macular thickness at first, CMT T1 = central macular thickness after first injection, CMT T3 = central macular thickness after 3 injections, CMT Tf = central macular thickness after one year, CRVO = central retinal vein occlusion, SD = standard deviation.			

**Fig. 2:**
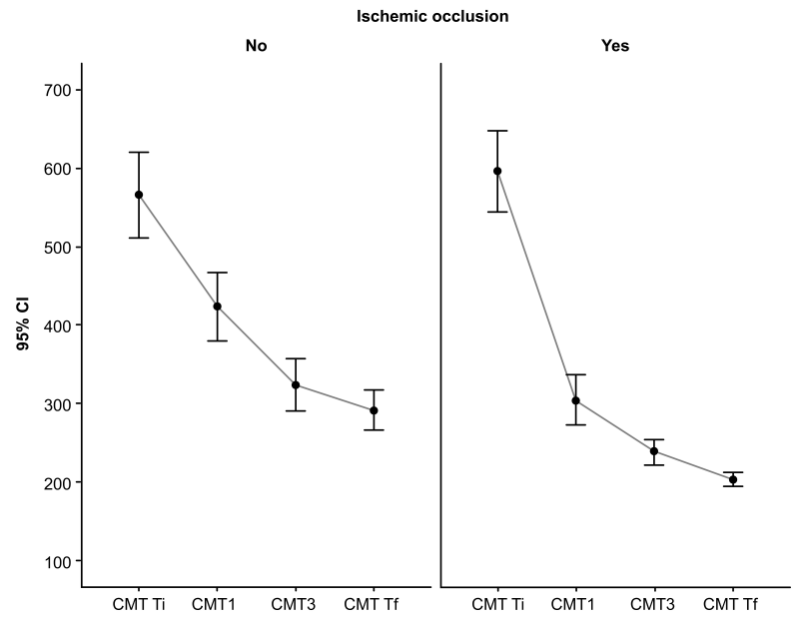
CMT decrease under treatment: greatest CMT decrease during first 3 injections, maintained after 12 months

 Overall, the difference in the evolution of CMT is statistically significant between ischemic and non-ischemic retinal vein occlusions (ANOVA test, F = 8.95, p = 0.04 < 0.05), with the best therapeutic response after the first injection (**Fig. 2**).

BRVO

 Analyzing the 26 cases of BRVO, macular dynamics were much more modest from the first evaluation to the 1-year check-up. The mean CMT at first was 424.8462 ±191.0308 in comparison with 566.0556 ± 199.8242 (non-ischemic CRVO) or 596.6129 ± 140.8036 (ischemic CRVO). Nevertheless, after 1 year of treatment, the mean CMT (257.8077 ± 17.1721) was comparable with the mean CMT in the CRVO group (273.5185 ± 191.6418).

**Fig. 3:**
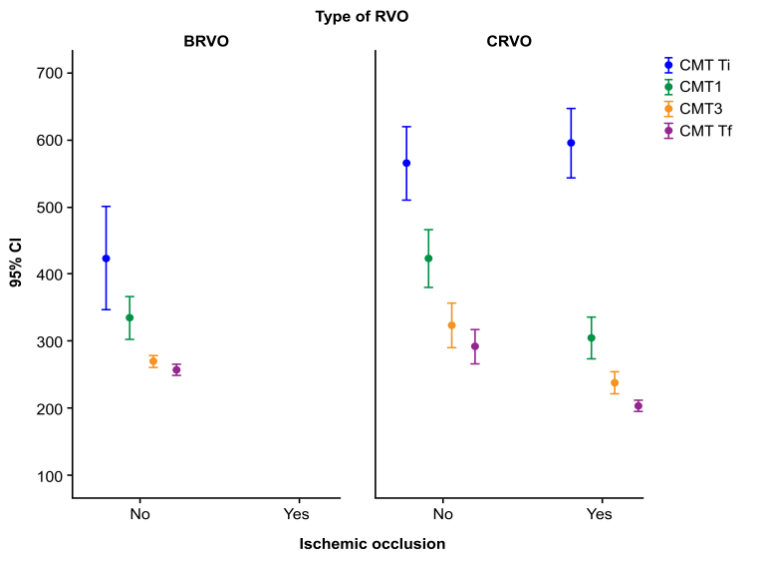
Evolution of CMT in BRVO cases compared to CRVO groups

Moreover, judging from the evolutive point of view, statistically significant differences were registered both between BRVO cases and non-ischemic CRVO ones (ANOVA test, F = 5.388, p = 0.000 < 0.05) and between BRVO cases and ischemic CRVO ones (ANOVA test, F = 25.193, p = 0.000 < 0.05) (**Fig. 3**).

This study revealed that no matter the type of occlusion, the most significant decrease was registered after the first injection, and the smallest difference was after the application of three injections, emphasizing the importance of prompt and sustained treatment from the very beginning. The greatest CMT variations were registered in ischemic CRVO, while the smallest ones were observed in the BRVO group, as expected.

 Connecting the CMT decrease with BCVA improvement, we did not register a statistically significant correlation in CRVO cases, meaning that no correlation between the CMT decrease and BCVA increase could be established in non-ischemic CRVO cases (R = 0.134, p = 0.472 > 0.05) and ischemic CRVO cases (R = 0.063, p = 0.651 > 0.05). Nevertheless, taking into consideration BRVO cases, a strong statistically significant correlation was found between these two variables (R = 0.633, p = 0.001 < 0.05; Evolution of CMT= 0.365 + 0.001 * Evolution of BCVA). This raises the assumption that other factors regarding macular dynamics and visual acuity prognosis could be much more valuable than CMT in patients with retinal vein occlusions.

*CMT and Posterior Vitreous Detachment (PVD)*

• BCVA increasing depending on PVD 

The increase in BCVA in the BRVO group of cases did not differ statistically significantly (Mann-Whitney Test, U = 60.000, p = 1.00 > 0.05) between cases with associated PVD and cases without associated PVD, while in the CRVO group it did (ANOVA test, F = 17.225, p = 0.000 > 0.05). The mean increase in logMAR visual acuity was 0.4685 ± 0.1232 l in BRVO cases with PVD and 0.7614 ± 0.5910 in BRVO cases without PVD. The mean increase in logMAR visual acuity was 1.4295 ± 0.4807 in CRVO cases without PVD and 0.9393 ± 0.5140 in CRVO cases with PVD.

• CMT decrease under intravitreal treatment depending on PVD

The decrease in CMT in the BRVO group of cases did not differ statistically significantly (ANOVA test, F = 3.247, p = 0.084 > 0.05), while in the CRVO group, it did (ANOVA test, F = 6.166, p = 0.015 < 0.05). The mean decrease in CMT was 131.20 ± 172.733 in BRVO cases without PVD and 286.50 ± 226.20 in BRVO cases with PVD. The mean decrease in CMT was 347.14 ± 171.47 in CRVO cases without PVD and 240.00 ± 197.56 in CRVO cases with PVD. It should be noted that the mean number of intravitreal injections was 5.20 for the BRVO group without PVD and 5.00 for the BRVO group with PVD, 6.88 for the CRVO group without PVD and 6.50 for the CRVO group with PVD.

Comparing CMT Ti in CRVO cases without PVD and those with associated PVD, the mean CMT Ti for the first group was 600.1475 ± 163.5592 and 518.875 ± 209.6135 for the second group. Moreover, the mean CMT Tf in CRVO cases without PVD and those with associated PVD was 253.0000 ± 86.0968 for the first group and 278.8750 ± 89.50653 for the second one. Although no statistically significant differences were observed at first (Mann-Whitney test, U = 566.000, p = 0.105 > 0.05) and after one year (Mann-Whitney test, U = 563.500, p = 0.100 > 0.05), in evolution, the decrease in CMT differed statistically between groups with PVD and those without.

*Different Macular Features-Ways to Connect with Visual Acuity Outcomes*

**Fig. 4:**
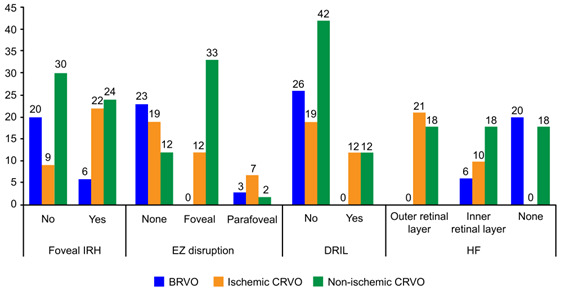
Macular features and distribution depending on the type of occlusion

 Several macular features were studied separately, depending on the type of occlusion and sometimes, where needed, depending on location. Therefore, foveal intraretinal hemorrhage was assessed as being present or not, with most cases being found in the CRVO group, no matter the type. For the ischemic type of occlusion, 70.96% of cases were associated with foveal IRH and only 29.03% were not. In BRVO cases, 76.93 percent of the patients did not present foveal IRH. All foveal EZ disruptions were found in the CRVO type of occlusion, while most cases with no EZ disruption were found in the BRVO type of occlusion. Few cases with retinal inner layer disorganization were registered among patients with CRVO. Overall, 53.42% of cases with HF were associated with outer retinal layer HF, all were registered with a CRVO type of occlusion, and 41.09% were associated with an inner retinal layer location, distributed among all three types of occlusion. Cases with no associated HF were split between the BRVO group and the non-ischemic CRVO group (**Fig. 4**).

*Foveal Intraretinal Hemorrhage*

 This feature is considered to be related to a poor visual acuity outcome. Our study revealed statistically significant differences in the BRVO group, analyzing BCVA evolution regarding the presence or absence of foveal intraretinal hemorrhage (Mann-Whitney Test, U = 24.00, p = 0.026 < 0.05), and also statistically significant differences both in the non-ischemic CRVO group (Mann-Whitney Test, U = 99, p = 0.000 < 0.05) and the ischemic CRVO group (Mann-Whitney Test, U = 54, p = 0.041 < 0.05) between cases with foveal intraretinal hemorrhage and cases without this macular feature.

Taking into consideration BRVO cases, from 26 of them, only 6 were associated with IRH. Cases with no IRH registered a mean increase of 0.4334 ± 0.1532 in logMAR BCVA and those with associated IRH registered a mean increase of 0.8785 ± 0.4628 in logMAR BCVA, which was considered statistically significant. In the non-ischemic CRVO group of patients, 30 patients were not associated with foveal intraretinal hemorrhage, while 24 patients presented with this macular feature. The difference in the mean increase in visual acuity was much more significant in non-ischemic CRVO cases with no IRH (0.7764 ± 0.41134) than in those with associated foveal IRH (1.3361 ± 0.3573). Ischemic CRVO cases included 9 patients without foveal intraretinal hemorrhage and 22 patients with foveal intraretinal hemorrhage. A very small improvement in logMAR BCVA was registered in this case, no matter the association with intraretinal hemorrhage (1.8997 ± 0.1532 without an association with foveal IRH and 1.6948 ± 0.2332 with an association with foveal IRH), although a statistically significant difference was observed between the groups.

Moreover, the number of intravitreal injections did not differ statistically regarding the presence or absence of foveal IRH in the BRVO group (Mann-Whitney Test, U = 54.000, p = 0.429 > 0.05) and the CRVO group (ANOVA test, F = 0.018, p = 0.895 > 0.05).

*Ellipsoid Zone Disruption*

Statistically significant differences were noted only for the evolution of visual acuity in non-ischemic CRVO cases, in correlation with the presence of ellipsoid zone disruption presence (Mann-Whitney Test, U = 45, p = 0.000 < 0.05). No statistically significant differences were noted for the BRVO group (Mann-Whitney Test, U = 33, p = 0.903 > 0.05) and ischemic CRVO cases (Mann-Whitney Test, U = 93.00, p = 0.373 > 0.05).

From 57 cases with EZ disruption, 12 were identified as parafoveal EZ disruptions and 45 as foveal EZ disruptions with statistically significant differences between the two groups (Mann-Whitney test, U = 22.500, p = 0.000 < 0.05). The better logMAR BCVA was correlated with a parafoveal location of the EZ disruptions (0.5816 ± 0.0880) compared with a foveal location of the EZ disruptions (1.3469 ± 0.4249). Most cases with foveal presence (33 out of 45) were registered among non-ischemic CRVO cases, while most cases of foveal presence (7 out of 12) were registered among ischemic CRVO cases.

Comparing patients with parafoveal EZ disruptions and those without EZ disruptions from all types of retinal vein occlusions, no significant statistical differences were noted regarding the evolution of logMAR BCVA (Mann-Whitney test, U = 268.500, p = 0.352 > 0.05), but statistically significant differences were noted when comparing cases with foveal EZ disruptions and those without EZ disruption for non-ischemic CRVO patients only (Mann-Whitney test, U = 9.000, p = 0.000 < 0.05).

*Hyperreflective Foci (HF)*

Judging the position of intraretinal hyper-reflective foci, every type of occlusion was devised regarding the association with the outer retinal layer HF, inner retinal layer HF, or no associated HF.

The BRVO group of cases included 20 cases with no associated HF and 6 cases of inner retinal layer HF, with statistically insignificant evolution of BCVA between the two groups (Mann-Whitney Test, U = 36.000, p = 0.139 > 0.05). The mean improvement in logMAR BCVA in association with inner retinal layer HF was 0.3785 ± 0.0848, in comparison with 0.5834 ± 0.3404 without associated HF. These results reflected that the inner retinal layer HF did not have a negative impact on visual acuity outcomes regarding BRVO cases.

Concerning the non-ischemic CRVO group, 18 patients were found in each of the three categories mentioned above, with statistically significant differences between them regarding logMAR BCVA evolution (Kruskal-Wallis Test, X2 = 29.275, p = 0.000 < 0.05). Comparing cases of no HF association with the inner retinal layer HF association group, no statistically significant evolution of BCVA was registered (Mann-Whitney Test, U = 160.50, p = 0.962 > 0.05). Mean logMAR BCVA improvement was 0.7524 ± 0.2951 for the first group and 0.7908 ± 0.4244 for the second, and therefore we could note similar evolutions. Nevertheless, comparing cases of no HF association with the outer retinal layer HF association group, a statistically significant evolution of BCVA was registered (Mann-Whitney Test, U = 0.00, p = 0.00 < 0.05). Mean logMAR BCVA improvement was 0.7524 ± 0.2951 for the first group and 1.5323 ± 0.1746 for the second, and therefore we could note very different kinds of evolution, proving outer retinal layer hyper-reflective foci to be a prognosis factor for poor visual acuity.

 Taking into consideration the ischemic CRVO group, 10 cases presented no HF (with a mean logMAR BCVA improvement of 1.5620 ± 0.1880), and 21 presented outer retinal layer HF (with mean logMAR BCVA improvement of 1.8458 ± 0.1907), with statistically significant differences between the groups regarding the evolution of BCVA (Mann-Whitney Test, U = 27.00, p = 0.01 < 0.05).

*Retinal Inner Layer Disorganization (DRIL)*

The disorganization of the retinal inner layer structure was registered only in CRVO, with 12 cases of non-ischemic-type CRVO and 12 cases of ischemic-type CRVO. Comparing visual acuity in non-ischemic CRVO cases with and without retinal inner layer disruption, no statistically significant differences were found (Mann-Whitney Test, U = 243.00, p = 0.851 > 0.05), and the same was true for ischemic CRVO cases (Mann-Whitney Test, U = 93.00, p = 0.373 > 0.05).

## Discussion

Our study underlined the fact that younger age was related to better visual acuity prognosis under the same kind of treatment and type of occlusion. Statistically significant differences were noted between patients under 40 years old and those over 60 years old and between patients aged 40-60 years and those over 60 years old. No statistically significant differences were noted between patients under 40 years old and those between 40 and 60 years old. Our results were similar in conclusions to other studies [**8**,**11**,**12**]. Meanwhile, in contrast to some studies, among our patients, women registered a better gain in visual acuity in all three age groups. Taking into consideration gender, a better gain in visual acuity was registered among women, no matter the age, although this was not statistically significant. Male cases registered no difference in logMAR BCVA evolution when comparing groups of age, while women registered significant differences between groups of age. A possible explanation for this could be the fact that among women, more cases of BRVO were registered (15 cases) than among men (11 cases), and the fact that men registered many more cases of ischemic CRVO (19 cases) than women (12 cases). This could be explained by the fact that cases with BRVO are known to have the best visual acuity prognosis, while CRVO cases are known to have the worst visual acuity prognosis. Moreover, the mean age of the women was registered as 63.53 years old, meanwhile the mean age of the men was registered as 68.79 years old. Additionally, the mean logMAR baseline BCVA among women was 0.73423 and only 0.91214 among men. Young age and better baseline BCVA were considered as factors indicating good prognosis, and these aspects provided a second explanation for our study results. 

Connecting a decrease in CMT with BCVA improvement, we did not register a statistically significant correlation in CRVO cases, meaning that no correlation between a CMT decrease and BCVA increase could be made concerning central venous retinal occlusions. This confirmed the assumption that other factors regarding macular dynamics and visual acuity prognosis could be much more valuable than CMT. Scott et al. [**12**] stated similar conclusions, which underlined the fact that a weak connection between these two variables could be made, and Tah et al. [**13**] sustained the fact that many other factors will have an impact on BCVA independently of CMT and could dissipate the dependence of this correlation [**13**,**14**]. Nevertheless, our results were different regarding the BRVO group of cases, in which a strong correlation was found between a CMT decrease and a BCVA increase. A reason for this result could be the fact that other factors that proved to have a strong impact on BCVA evolution and CMT decrease in our study were not or were weakly represented in the BRVO group (for example, no registered outer retinal layer disruption cases, no foveal EZ disruption cases and only six cases with foveal IRH). The absence or weak representation of these macular features will offer space for a better correlation between BCVA increase and CMT decrease. 

Several studies demonstrated the presence of foveal intraretinal hemorrhage as a macular feature being a predictive factor for poor visual outcome [**15**]. We could also confirm this, with the specification that this result was similar in the CRVO group of patients and the BRVO group. According to Goerlitz-Jessen et al. [**16**], the presence of foveal IRH in patients with CRVO was associated with a poorer response to intravitreal treatment and therefore a need for more intravitreal injections over time. Judging from the same perspective, in our study, we found that for a similar (not statistically significant) number of injections applied to both groups, patients with foveal intraretinal hemorrhage registered a poorer retinal response. The inter-correlation of anti-VEGF therapy and foveal IRH regarding retinal vein occlusions was not yet very well clarified compared to other pathologies (for example diabetic retinopathy), and further studies regarding the exact pathophysiological mechanism and the way it responds to several substances need to be conducted. Previous studies [**17**,**18**] stated that the presence of foveal-involving retinal hemorrhage will have an impact on foveal ellipsoid zone integrity and external limiting membrane integrity, factors that proved to have a negative impact on visual acuity outcome. Our study showed a strong correlation between cases with no foveal intraretinal hemorrhage and those with no foveal EZ disruption and also between cases with foveal intraretinal hemorrhage and foveal EZ disruption in non-ischemic CRVO cases. 

Our study revealed that only outer retinal layer hyper-reflective foci can be considered as predictive factors for poor visual acuity outcome, with the association of inner retinal layer HF, showing no statistically significant differences concerning the mean evolution of BCVA. Similar results were described in a detailed manner by Mo et al. [**19**] in their study in 2017. There are many theories concerning the origin and components of hyper-reflective foci in several retinal pathologies, for example, diabetic macular edema, age-related macular degeneration, or retinal vein occlusions, but the most significant conclusion is that their presence is correlated with retinal inflammation [**18**,**20**]. Previous studies have demonstrated the migration of HF from the inner retinal layers to the outer retinal layers as a sign of the continuous inflammatory condition of the retina, underlying the need for associated steroid intravitreal injection in these cases. The explanation for the poor visual acuity outcome in association with outer retinal layer HF was not simply their presence, but rather the prolonged inflammatory condition of the retina associated with HF’s presence and migration to the inner retinal layers. This aspect fortified the idea that HF is a guiding factor for the use of intravitreal steroids alongside anti-VEGF treatment, no matter the risk of cataract development. 

Previous studies underlined the fact that large areas of disruption of the ellipsoid zone were associated with poorer visual acuity outcomes. Additionally, higher CMT due to macular edema with a weak response to intravitreal treatment was associated with the disruption of the photoreceptor integrity, which is an essential part of the visual pathway and can be visualized as a disintegration of the EZ on SD-OCT. Our study compared visual acuity evolution regarding this macular feature and observed statistically significant differences only in the non-ischemic CRVO group of patients. Cases with BRVO and ischemic CRVO did not evolve differently in association with the disruption of the EZ [**7**,**21**]. Moreover, separating the patients with EZ disruptions in foveal and parafoveal areas, we concluded that statistically significant differences were found between these two groups regarding logMAR BCVA evolution. Nevertheless, comparing cases with a parafoveal location of EZ disruption and cases of no EZ disruption, no statistically significant differences were registered. Furthermore, since our lot of patients presented so many cases with foveal EZ disruptions in the non-ischemic CRVO group, we were able to compare these patients with cases of no EZ disruptions in the same type of occlusion, the non-ischemic CRVO group, and statistically significant differences were registered. Therefore, the presence and location of EZ disruption, alongside the area of EZ disruption, are very important for an accurate prognosis, but we can assume (taking into consideration our study results) that only foveal EZ disruption may be a factor of poor visual acuity prognosis.

Yin et al. [**7**], together with other previous authors, described a strong correlation between DRIL and visual acuity outcome, underlining their direct connection. Still, in our study, we judged this macular feature’s role based on BCVA evolution, and no statistically significant results were found. Therefore, we could not establish this aspect as a predictive one in the visual acuity outcomes [**7**].

Waldstein et al. [**22**] stated in their study that in the CRVO group, eyes with complete PVD showed a higher mean baseline BCVA than eyes without PVD, while baseline BCVA was similar in eyes with and without PVD in the BRVO group. Our study came to the very same conclusion, judging upon the evolution of BCVA, no matter the type of occlusion. Still, taking into consideration the CMT decrease, things have proved to be different between the BRVO group and the CRVO one. Among BRVO cases, a higher decrease in CMT was registered in association with complete PVD than without association with PVD, after a similar number of intravitreal injections. Among CRVO cases, a higher decrease in CMT was registered in cases without PVD than in those with complete PVD, after a similar number of intravitreal injections. Going further with the analysis, we found no statistically significant difference between CMT Ti and CMT Tf regarding the association of PVD in CRVO cases. Nevertheless, higher CMT Ti was observed among patients without PVD in comparison with CMT Ti among patients with PVD, although statistically insignificant. When analyzing the final assessment, the results were the exact opposite. This could be the explanation for a different evolutive line between the two groups. 

There were several limitations to our study. One of these limitations was the impossibility of evaluating each patient in the same period from the moment of the occlusion because of the very different moments chosen by the patients for visiting an eye specialist. Secondly, we were unable to treat patients with other anti-VEGF medication due to higher out-of-pocket costs. Also, we used an individualized intravitreal treatment plan, guided by the grade of macular edema and neovascularization. Thirdly, we did not analyze macular features (for example, foveal IRH, HF, or EZ disruption) from a quantitative point of view.

## Conclusions

 The most important non-imaging predictive factors regarding visual acuity after retinal vein occlusions were age and visual acuity at the moment of the patient’s first visit to an ophthalmologist. CMT dynamics have a weak connection with visual acuity fluctuations in the CRVO group of cases and a much better correlation in the BRVO group of cases. The presence of foveal IRH, outer retinal layer HF, and foveal EZ disruption has a negative impact on visual acuity outcomes. Parafoveal EZ disruption may not have an impact on visual acuity outcome or inner retinal layer features (disorganization or HF). 

**Conflict of Interest statement**

The authors state no conflict of interest.

**Informed Consent and Human and Animal Rights statement**

Informed consent has been obtained from all individuals included in this study.

**Authorization for the use of human subjects**

Ethical approval: The research related to human use complies with all the relevant national regulations and institutional policies, is by the tenets of the Helsinki Declaration, and has been approved by the Ethics Committee of “Victor Babeş” University of Medicine and Pharmacy, Timişoara, Romania.

**Acknowledgments**

None.

**Sources of Funding**

None.

**Disclosures**

None.

## References

[1] Rogers SL, McIntosh RL, Lim L, Mitchell P, Cheung N, Kowalski JW (2010). Natural history of branch retinal vein occlusion: an evidence-based systematic review. Ophthalmology.

[2] Woo SC, Lip GY, Lip PL (2016). Associations of retinal artery occlusion and retinal vein occlusion to mortality, stroke, and myocardial infarction: a systematic review. Eye (Lond).

[3] Blair K, Czyz CN (2023). Central retinal vein occlusion.

[4] Kida T (2017). Mystery of retinal vein occlusion: vasoactivity of the vein and possible involvement of endothelin-1. Biomed Res Int.

[5] Hayreh SS, Zimmerman MB, Podhajsky P (1994). Incidence of various types of retinal vein occlusion and their recurrence and demographic characteristics. Am J Ophthalmol.

[6] Poh S, Tham YC, Chee ML, Dai W, Majithia S, Soh ZD (2020). Association between macular thickness profiles and visual function in healthy eyes: the Singapore epidemiology of eye diseases (SEED) study. Sci Rep.

[7] Yin S, Cui Y, Jiao W, Zhao B (2022). Potential prognostic indicators for patients with retinal vein occlusion. Front Med (Lausanne).

[8] Daien V, Navarre S, Fesler P, Vergely L, Villain M, Schneider C (2012). Visual acuity outcome and predictive factors after bevacizumab for central retinal vein occlusion. Eur J Ophthalmol.

[9] Arrigo A, Aragona E, Lattanzio R, Scalia G, Bandello F, Parodi MB (2021). Collateral vessel development in central and branch retinal vein occlusions are associated with worse visual and anatomic outcomes. Invest Ophthalmol Vis Sci.

[10] Fragiotta S, Abdolrahimzadeh S, Dolz-Marco R, Sakurada Y, Gal-Or O, Scuderi G (2021). Significance of hyperreflective foci as an optical coherence tomography biomarker in retinal diseases: characterization and clinical implications. J Ophthalmol.

[11] Li Y, Hall NE, Pershing S, Hyman L, Haller JA, Lee AY (2022). Age, gender, and laterality of retinal vascular occlusion: a retrospective study from the IRIS® registry. Ophthalmol Retina.

[12] Scott IU, VanVeldhuisen PC, Oden NL, Ip MS, Blodi BA, Jumper JM (2009). SCORE study report 1: baseline associations between central retinal thickness and visual acuity in patients with retinal vein occlusion. Ophthalmology.

[13] Tah V, Orlans HO, Hyer J, Casswell E, Din N, Shanmuganathan VS (2015). Anti-VEGF therapy and the retina: an update. J Ophthalmol.

[14] Sasajima H, Zako M, Maeda R, Murotani K, Ishida H, Ueta Y (2022). Foveal intraretinal fluid localization affects the visual prognosis of branch retinal vein occlusion. J Clin Med.

[15] Powers JH, Thomas AS, Mir TA, Kim JS, Birnbaum FA, Yoon SP (2019). Impact and implication of fovea-involving intraretinal hemorrhage after acute branch retinal vein occlusion. Ophthalmol Retina.

[16] Goerlitz-Jessen M, Mir TA, Thomas AS, Yoon SP, Fekrat S (2018). The impact and implication of a fovea-involving intraretinal hemorrhage in patients with central retinal vein occlusion. Invest Ophthalmol Vis Sci.

[17] Hendrick A, VanVeldhuisen PC, Scott IU, King J, Blodi BA, Ip MS (2021). SCORE2 report 13: intraretinal hemorrhage changes in eyes with central or hemiretinal vein occlusion managed with aflibercept, bevacizumab or observation. Secondary analysis of the SCORE and SCORE2 clinical trials. Am J Ophthalmol.

[18] Muraoka Y, Tsujikawa A, Takahashi A, Iida Y, Murakami T, Ooto S (2015). Foveal damage due to subfoveal hemorrhage associated with branch retinal vein occlusion. PLoS One.

[19] Mo B, Zhou HY, Jiao X, Zhang F (2017). Evaluation of hyperreflective foci as a prognostic factor of visual outcome in retinal vein occlusion. Int J Ophthalmol.

[20] Deb AK, Sarkar S (2021). Commentary: hyperreflective foci on optical coherence tomography and their clinical implications in diabetic macular edema. Indian J Ophthalmol.

[21] Etheridge T, Dobson ETA, Wiedenmann M, Oden N, VanVeldhuisen P, Scott IU (2021). Ellipsoid zone defects in retinal vein occlusion correlates with visual acuity prognosis: SCORE2 report 14. Transl Vis Sci Technol.

[22] Waldstein SM, Montuoro A, Podkowinski D, Philip AM, Gerendas BS, Bogunovic H (2017). Evaluating the impact of vitreomacular adhesion on anti-VEGF therapy for retinal vein occlusion using machine learning. Sci Rep.

